# Primary schools and the amplification of social differences in child mental health: a population-based cohort study

**DOI:** 10.1136/jech-2017-208995

**Published:** 2017-10-22

**Authors:** Louise Marryat, Lucy Thompson, Helen Minnis, Philip Wilson

**Affiliations:** 1 Farr Institute, Scottish Collaboration for Public Health Research and Policy, University of Edinburgh, Edinburgh, UK; 2 Institute of Health and Wellbeing, University of Glasgow, Glasgow, UK; 3 Centre for Rural Health, University of Aberdeen, Aberdeen, UK

**Keywords:** psychology, psychiatry, mental health, child health, life course epidemiology

## Abstract

**Background:**

This paper examines socioeconomic inequalities in mental health at school entry and explores changes in these inequalities over the first 3 years of school.

**Methods:**

The study utilises routinely collected mental health data from education records and demographic data at ages 4 and 7 years, along with administrative school-level data. The study was set in preschool establishments and schools in Glasgow City, Scotland. Data were available on 4011 children (59.4%)at age 4 years, and 3166 of these children were followed at age 7 years (46.9% of the population). The main outcome measure was the teacher-rated Goodman’s Strengths and Difficulties Questionnaire (4–16 version) at age 7 years, which measures social, emotional and behavioural difficulties.

**Results:**

Children living in the most deprived area had higher levels of mental health difficulties at age 4 years, compared with their most affluent counterparts (7.3%vs4.1% with abnormal range scores). There was a more than threefold widening of this disparity over time, so that by the age of 7 years, children from the most deprived area quintile had rates of difficulties 3.5 times higher than their more affluent peers. Children’s demographic backgrounds strongly predicted their age 7 scores, although schools appeared to make a significant contribution to mental health trajectories.

**Conclusions:**

Additional support to help children from disadvantaged backgrounds at preschool and in early primary school may help narrow inequalities. Children from disadvantaged backgrounds started school with a higher prevalence of mental health difficulties, compared with their more advantaged peers, and this disparity widened markedly over the first 3 years of school.

## Introduction

The Marmot review argued that ‘Giving every child the best start in life is crucial to reducing health inequalities across the life course. The foundations for virtually every aspect of human development—physical, intellectual and emotional—are laid in early childhood’.^1^ There is an increasing recognition of the impact of early inequalities on health outcomes.^2^

Mental health disorders encompass a range of symptoms and behaviours that cause substantial distress or interfere with personal functioning. In childhood, these frequently revolve around emotional disorders, such as anxiety, depression and obsessions; conduct disorders, often including troublesome, aggressive or antisocial behaviours; and hyperactivity disorders that encompass both inattention and hyperactivity.^3^ Around 1 in 10 children in the UK experience a mental health disorder,^4^ with a lifetime prevalence of 1 in 4.^5^ Even among children who receive a formal mental health diagnosis, only 10% of 4-year olds and 25% of adolescents with a diagnosis are likely to receive a service.^6^

Children with mental health problems experience a range of difficulties throughout childhood, including poorer physical health and problems in forming relationships and in fully participating in school and home activities.^7^ Mental health disorders in childhood are associated with a range of future negative outcomes, including poorer academic achievement, future mental health problems, increased criminality, risky sexual behaviours, poorer relationships and unemployment.^8–13^

Socioeconomic differences in mental health have been seen from an early age, with poverty as a key associated factor.^4 14–16^ Adversity in the first year of life has been associated with externalising problems at age 3 years, and adversity at ages 2–3 years predicts both externalising and internalising difficulties at age 3 years.^17 18^ Cohort studies of this type generally rely on parent-reported data, which can be influenced by the parent’s own mental health.^19^ To date, there has been little research on disparities in childhood mental health, with even a review paper on this topic only highlighting the impact of poverty rather than inequality per se.^20^ Early intervention to reduce mental health inequalities could benefit disadvantaged children and society as a whole.

This paper explores the socioeconomic inequalities in teacher-rated mental health^i^ in the early years, and how this changes during the first few years of school, using data collected in Glasgow City, Scotland.

Research questions:

1. What disparities are evident between socioeconomic groups in mental health difficulties in preschool and primary school?
2. What happens to these disparities over the first 3 years of primary school?
3. Can mental health trajectories be explained by (1) children’s demographics at school entry and (2) school characteristics, such as denomination?

## Methods

The key outcome measure in the study was Goodman’s Strengths and Difficulties Questionnaire (SDQ).^21^ The SDQ is a brief behavioural screening questionnaire containing 25 questions covering five domains: Conduct Problems, Hyperactivity/Inattention, Emotional Symptoms, Peer Relationship Problems and Prosocial Skills. The first four scales can be combined to create a Total Difficulties score. There are several versions of the SDQ available: the current study used the age 3–4 teacher-rated version in preschool (at age 4 years) and the age 4–16 teacher-rated version in Primary 3 (at age 7–8 years). The two age versions are similar to each other and bar two items in the Conduct Problems scale, which are ‘softer’ in the version of ages 3–4 years to reflect children’s development at that age. A cut-off (children scoring above 16/40)^ii^ is available, which produces an ‘abnormal’ score, giving an idea of children with likely difficulties. This cut-off has been established to include approximately 10% of children aged 4–16 years in a normal population.^22^ Although models fitted using the continuous scores would carry more power, the current study uses the banded scores as they were felt to be more meaningful to both education and health professionals using the results.

It is important to note that the SDQ does not give a diagnosis of particular mental health disorders but rather gives a broad idea of mental health difficulties. The scale directly measures some aspects of mental health, such as symptoms of hyperactivity and inattention, and indirectly measures others, such as having frequent tummy aches or headaches and frequently being worried, which may both indicate emotional disorders such as anxiety in children. Furthermore, there are some mental health disorders that are not covered in the SDQ, such as eating disorders and selective mutism, although it is likely that such disorders are comorbid with other mental health disorders.^23^

The key measure of deprivation was the Glasgow Index of Multiple Deprivation (GIMD). This is based on the Scottish Index of Multiple Deprivation (SIMD), which is renewed every 4 years and calculated using measures of income, housing, crime, employment, health and education data.^24^ Due to Glasgow’s relatively high levels of deprivation, this study used ‘Glasgow SIMD quintiles’ (GIMD) for these analyses, which group the SIMD scores into quintiles within Glasgow City only, leading to similar numbers of individuals in each quintile.^iii^ All variables are described in table 1.

**Table 1:** Description of variables explored in the analysis

Level	Variable	Source
Individual	Preschool mental health difficulties	Teacher-rated SDQ—Education Services routine data
	Sex	Education Services routine data
	Ethnicity	Education Services routine data
	Glasgow Index of Multiple Deprivation (at preschool and Primary 3)	From child’s home postcode, which is collected as part of Education Services routine data
	Looked After Status (at preschool and Primary 3)	Education Services routine data
School	Religious affiliation (denominational or non-denominational)	Education Scotland
	Her Majesty’s Inspectorate for education (HMIe)/Education Scotland follow-up report required (where the initial school report was unsatisfactory)	Education Scotland
	School pupil roll	Schools census—Education Services routine data
	School exclusions per 1000 pupils	Schools census—Education Services routine data
	Proportion of children entitled to free school meals (a means-tested benefit when collected)	Schools census—Education Services routine data

SDQ, Strengths and Difficulties Questionnaire.

Formal ethical review was not required for this study because of it being an analysis of secondary data, which were collected as part of a service review. Data were anonymised prior to use for research purposes. Although formal ethical review board approval was not required for the present analysis of the data, the ethical issues possibly raised by this study were considered by the research team. It was concluded that the project posed no harm to the participants, the schools or the different regions, as the anonymised data were collected by educational establishments as part of the routine documentation passed to primary schools for the benefit of teachers and pupils. For example, teachers are trained to interpret results and are encouraged to use them to flag up where children may need extra support in the early days of primary school. In addition, data are being used by Education Services at a higher level to explore issues such as the following: does nursery size affect social, emotional and behavioural development, which can then be taken into account when planning for new city nurseries.

Overall, 4011 children had a preschool (age 4 years) SDQ completed in 2012. This equates to 59.4% of the estimated preschool population in Glasgow City in that year (n=6756).^25^ Of these, 3166 were able to be matched with their age 7 SDQ (79%). This meant that we had matched preschool and Primary 3 data for 46.9% of the preschool population in 2012. Data at age 7 years were collected from all 137 state-funded primary schools in the city.

Multilevel models, using MLwiN V.2.18 to evaluate the mental health of children within schools, were fitted, predicting having an abnormal Total Difficulties score at age 7 years. This type of analysis is similar to standard regression modelling; however, it has the added advantage of taking into account different levels of grouping, for example, children within schools. This is important because, in this example, children within a school are more similar to each other than to children in another school (due to catchment areas, parent selection of school, etc). Because of this, using a standard regression model may mean that error is not adequately controlled for, in the way that multilevel models control for it. Empty single-level and two-level models (children within schools) were fitted first to explore whether multilevel models were necessary. The Wald statistic from the empty model suggested that there were significant differences in levels of abnormal Total Difficulties scores at age 7 years, and thus multilevel models should be fitted in order to give the correct errors in the models. Furthermore, residuals from the empty model were plotted: 10 schools (out of the 137) had 95% CIs, which lay above the ‘0’ line, indicating a statistically significant difference in these schools’ levels of abnormal scores compared with the mean, again supporting the need for multilevel (as opposed to single-level) models being fitted.

Each child level variable was individually entered into an unadjusted model in order to assess the significance of each factor at a binary level. Each of the level 2 (school-level) variables was then entered into multilevel models one at a time as fixed effects. The significant variables were then entered into the multivariate multilevel linear model in clusters by type of variable. Child characteristics, such as gender and ethnicity, were entered into the model first, followed by any significant family characteristics at the child level (eg, home area deprivation). Each variable was checked for statistical significance, and any variables that lost significance were removed one at a time from the model. Residuals from the adjusted model were again examined visually through caterpillar plots.

## Results

The sample demographics were similar to that of the population of children in Glasgow: 51.6% were girls, 28%–29% lived in the areas of highest deprivation within the city at each time point (using quintiles of GIMD), compared with 13% in the most affluent. Three-quarters (74.2%) of the children were of a white UK ethnicity, compared with 71% in the overall school-age population in 2014.^26^ In the cohort at preschool, 1.8% of children had ‘Looked After Status’ (meaning that they were under the supervision of Social Work Services, either living at home with their parent(s) or living away from home in various settings), rising to 2.8% at Primary 3. This compares with 3% in the population of Glaswegian children overall (table 2).

**Table 2:** Frequencies of cohort characteristics

Variable	% (n)
Sex	
Boys	48.4 (1939)
Girls	51.6 (2071)
Ethnicity	
White UK	74.2 (2312)
Non-white UK	25.8 (806)
Looked After Status at preschool	
Not ‘Looked After’	98.2 (3936)
‘Looked After’	1.8 (74)
Home multiple deprivation quintile (at preschool)	
1—Most deprived	28.4 (1104)
2	22.5 (876)
3	19.1 (743)
4	16.7 (648)
5—Least deprived	13.3 (517)
Looked After Status at Primary 3	
Not ‘Looked After’	97.2 (3898)
‘Looked After’	2.8 (112)
Home multiple deprivation quintile (at Primary 3)	
1—Most deprived	27.5 (1103)
2	23.8 (955)
3	22 (882)
4	16.7 (670)

Children who lived in the most deprived area quintile in Glasgow when they started school were substantially more likely to have an abnormal SDQ score at age 4 years, compared with children in the most affluent area quintile (7.3% vs 4.1%). Strikingly, by age 7 years, the gap between the two groups had widened 3.5-fold, so that 14.7% of children in the most deprived area quintile had an abnormal score, compared with 3.6% of children from the most affluent area quintile. Differences were even starker in boys: scores in the most deprived quintile rose from 11.5% at age 4 years to 20.1% at age 7 years, while in the most affluent quintile, scores fell from 6.7% to 5.3%.

**Figure 1:**
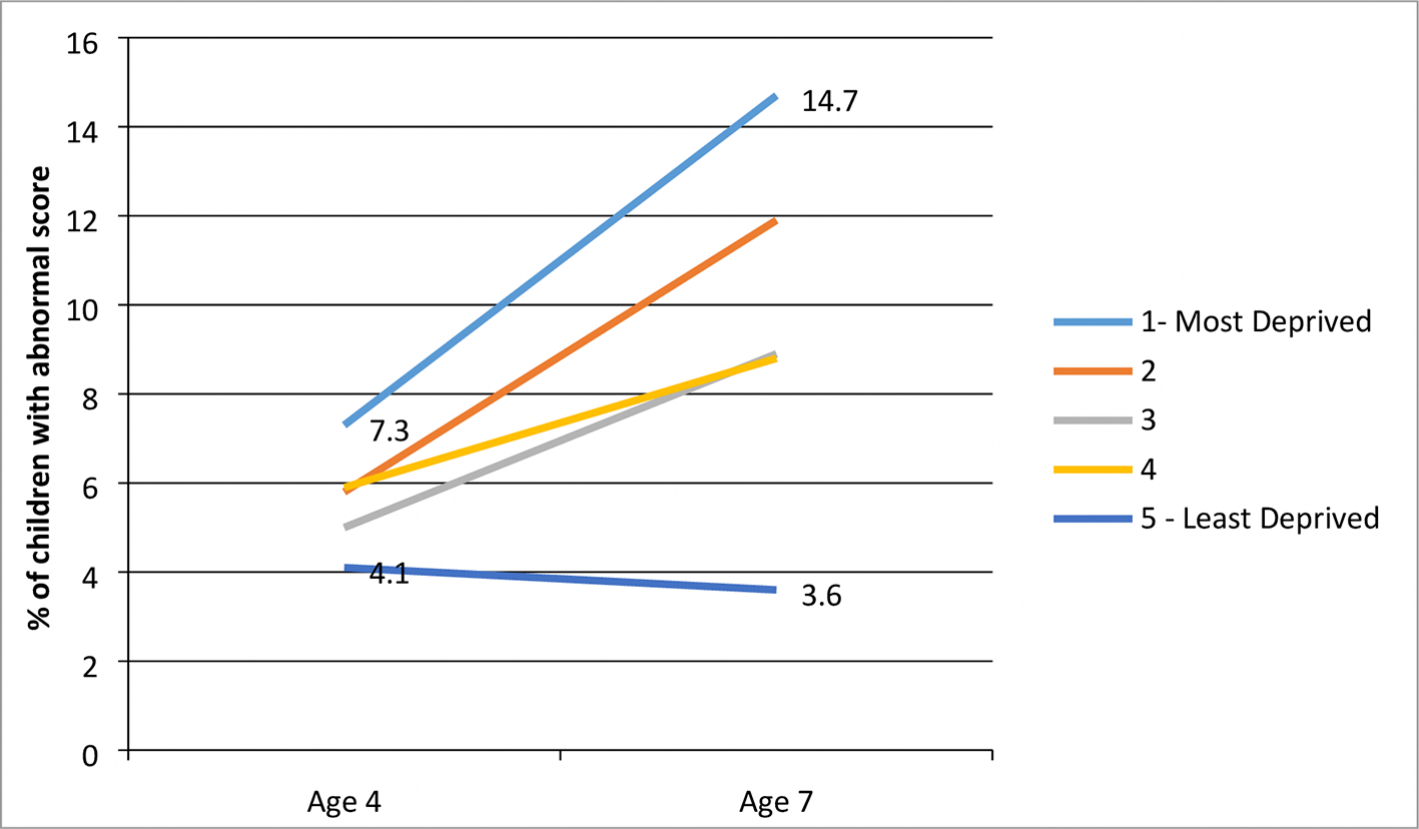
Proportion of children in the ‘abnormal’ total difficulties group by age and level of area deprivation (base: 3078).

The only group in which scores fell between preschool and Primary 3 was the most affluent: scores increased in all other socioeconomic groups (figure 1).

The disparity in mental health difficulties changed to different extents depending on which subscale of the SDQ was examined. The largest increase in inequalities was on the hyperactivity/inattention domain, where the prevalence levels between the least and the most deprived quintiles increased by almost 10%, from a difference of 4% at age 4 years to 13.8% at age 7 years, with the disparity in conduct problems also increasing by 5.5%. By contrast, peer relationship difficulties fell for all groups, with the gap remaining broadly similar (increasing by 0.8%), while the difference between levels of abnormal prosocial behaviour scores decreased by 0.6%, again with levels in all groups falling (table 3).

**Table 3:** Proportion of children with abnormal Total Difficulties* scores by age and deprivation at ages 4 and 7

	Age 4 years			Age 7 years			Difference in disparity between ages 4 and 7 years
	Most deprived	Least deprived	Disparity between most and least deprived quintiles	Most deprived	Least deprived	Disparity between most and least deprived quintiles	
Emotional Symptoms	5.8	3.3	2.5	7.8	3	4.8	2.3
Conduct Problems	8.2	5	3.2	11.7	3	8.7	5.5
Hyperactivity/Inattention	10.4	6.4	4	22.3	8.5	13.8	9.8
Peer Relationship Problems	9.1	5.6	3.5	6	1.7	4.3	0.8
Prosocial skills	13.7	7.9	5.8	10.2	5	5.2	−0.6

*Total Difficulties is the sum of the four negative SDQ scales: Conduct Problems, Hyperactivity/Inattention, Peer Relationship Problems and Emotional Symptoms.

Having established that there does appear to be a widening of inequalities between ages 4 and 7 years, a series of multilevel models were fitted to explore which factors independently predict having an abnormal score at age 7 years. At a univariable level, all individual-level variables were significantly associated with having an abnormal Total Difficulties score at age 7 years. The strongest predictor of having an abnormal score at age 7 years was having an abnormal Total Difficulties score at age 4 years (OR 6.05, 95% CI 5.86 to 6.24). Current (Primary 3) Looked After Status gave higher odds of an abnormal Total Difficulties score at age 7 years (OR 4.1, 95% CI 3.88 to 4.32), and level of home area deprivation gave higher odds at age 4 years (preschool) (OR for most deprived quintile 1.73, 95% CI 1.42 to 2.06) (table 4). These two variables were, therefore, put into to multivariable models, alongside the other individual-level variables.

**Table 4:** Unadjusted multilevel models: pupil-level variables

	Beta coefficient (SE)	OR
Sex		
Male	1.06 (0.13)**	2.89
Female	0	
Ethnicity		
White UK	0.95 (0.18)**	2.59
Non-white UK	0	
Preschool ever Looked After		
Ever Looked After	1.32 (0.32)**	1.74
Never Looked After	0	
Home GIMD (preschool)		
1—Most deprived	1.73 (0.56)**	5.64
2	1.41 (0.57)**	4.1
3	0.5 (0.65)	
4	0.5 (0.65)	
5—Least deprived	0	
Primary 3 Ever Looked After		
Ever Looked After	1.41 (0.22)**	4.1
Never Looked After	0	
Home GIMD (Primary 3)		
1—Most deprived	1.65 (0.55)**	5.21
2	1.31 (0.56)*	3.71
3	1.38 (0.58)*	3.97
4	1.38 (0.57)*	3.97
5—Least deprived	0	
Preschool SDQ Total Difficulties		
Abnormal	1.8 (0.19)*	6.05
Normal	0	

p Values: *<0.05; **<0.01.

GIMD, Glasgow Index of Multiple Deprivation; SDQ, Strengths and Difficulties Questionnaire.

At a univariable level, two variables that were operating at a school level were significantly related to having an abnormal score at Primary 3. A higher proportion of children within the school who were entitled to free school meals (a proxy for income levels of children within the school) were associated with higher levels of abnormal scores at age 7 years, as was having a higher number of school exclusions per 1000 pupils in a school. School religious affiliation, attendance levels and inspection report results were not associated with higher levels of abnormal scores (table 5).

**Table 5:** Univariable multilevel models containing school-level variables

	Beta coefficient (SE)	OR
Religious affiliation		
Catholic	0.14 (0.19)	–
Non-denominational	0	
Attendance		
Below average	0.13 (0.23)	–
Average or above	0	
Her Majesty’s Inspectorate for education/Education Scotland score (continuous)	−0.04 (0.12)	–
Follow-up inspection required (continous)†	−0.16 (0.18)	–
% FSM entitlement (continous)‡	0.021 (0.006)**	**1.02**
Exclusions per 1000 pupils (continous)§	0.005 (0.002)**	**1.01**

p Values: *<0.05; **<0.01.

†Where a school did not reach a satisfactory level during a school inspection, they may be required to have a follow-up inspection a short time later. These data, therefore, give an indication of an overall poor inspection report.

‡The proportion of children in a school who, due to their household income, are entitled to receive a free school meal (FSM).

§The number of pupils per 1000 in a school who have been excluded from school in the previous year.

School-level variables significant at a binary level were no longer significant once individual-level characteristics were controlled for; therefore, the final model predicting an abnormal Total Difficulties score at age 7 years contained only individual-level variables (table 6). The strongest predictor of having an abnormal score at age 7 years was having an abnormal score at age 4 years (OR 5.05, 95% CI 4.84 to 5.26). Both living in a more deprived area at age 4 years and having ever Looked After Status at age 7 years were associated with having an abnormal Total Difficulties score at age 7 years, as was being male and being of a white UK ethnicity.

**Table 6:** Final multivariable multilevel model predicting Total Difficulties abnormal score at age 7 years

	Beta coefficient (SE)	OR
Constant	−4.6 (0.36)**	
Sex		
Male	0.99 (0.14)**	2.69
Female	0	
Ethnicity		
White UK	0.82 (0.19)**	2.27
Non-white UK	0	
Home GIMD (preschool)		
1—Most deprived	1.16 (0.33)**	3.19
2	1.1 (0.33)**	3.0
3	0.74 (0.34)*	2.1
4	0.79 (0.34)*	2.2
5—Least deprived	0	
Primary 3 Ever Looked After		
Ever Looked After	1.29 (0.25)**	3.63
Never Looked After	0	
Preschool SDQ Total Difficulties		
Abnormal	1.62 (0.21)**	5.05
Normal	0	
Cons/cons	0.62 (0.15)	
Schools	137	
Cases	3033	

p Values: *<0.05; **<0.01.

GIMD, Glasgow Index of Multiple Deprivation; SDQ , Strengths and Difficulties Questionnaire.

Variables entered into the models prior to this were as follows: (level 1—child) sex, ethnicity, preschool GIMD, Primary 3 Looked After Status and preschool SDQ Total Difficulties; (level 2—school) percentage on free school meals and exclusions per 1000 pupils.

Although none of the measured school-level variables were independently associated with having an abnormal Total Difficulties score at age 7 years, there was a significant amount of variation between schools, which was unexplained. Nine schools (6.6%) had scores significantly higher than the mean, which is more than the 5%, which would be expected by chance.

## Discussion

It appears that the first 3 years of primary school amplify the already marked inequalities in mental health difficulties, and that some unmeasured school factors may play a role in this. In some schools, children had lower levels of mental health at age 7 years, even after their mental health score at preschool and intake characteristics were controlled for. School-level variance, which appeared to be an important factor in relation to the widening mental health disparity, was unexplained using the available measures: none of the school-level characteristics that were tested explained any of the variance in the models.

### Strengths

The major strength of this study is that it uses routinely collected data collated by Glasgow City Council Education Services: this ensures high completion rates and thus avoids the traditional cohort study problems of selective attrition based on behavioural problems and deprivation.^27^ The SDQ is a widely used and well-validated measure. The use of the teacher-rated version of the SDQ is arguably more objective than the parent-rated SDQs used in the majority of previous studies, the latter of which may be associated with the parent’s own mental health or their view of what ‘normal’ may be, rather than by comparison with peers.

### Weaknesses

The main strength of the study is also its weakness: the fact that we are dependent on routinely collected data, while having many advantages, also has the disadvantage of lack of detail. The study lacks many of the demographic variables, which may explain more of the individual-level variation (eg, lone parent status and household income), as well as the ‘softer’ school-level measures (eg, teacher–pupil relationships, school culture and environment). In addition, data were collected in Glasgow City alone. Glasgow City is a particularly deprived part of the UK and faces substantial issues with substance misuse and violence, as well as poor health across the life course. This may mean that socioeconomic inequalities have a greater effect here than in other parts of the world, although another study by the authors using Scottish birth cohort data indicated that mental health difficulties at the preschool stage were no different in other parts of Scotland.^28^

Differences by school in children’s mental health were found, even after children’s preschool mental health score and the intake characteristics of the school were controlled for. Such findings are supported by previous evidence, which suggests that school differences exist at the primary school age in relation to both victimisation and bullying^29 30^ and behavioural problems.^31 32^ Rutter suggested that most schools are ‘good enough’ for children’s development and that the quality of schools in the UK demonstrates little overall variation. He suggested that only around 3% of the variance is explained by the school, in contrast to 11% at the class level and 86% at the individual level.^31^ In the present study, after individual and school characteristics were controlled for, only 9 out of 137 schools were performing significantly worse in relation to student’s mental health. Future research is required to discover what is different about these schools; however, overall the results do suggest that the majority of schools are very similar in their levels of children’s mental health difficulties, once other factors are controlled for.

The strongest predictor of having mental health difficulties at age 7 years was having mental health difficulties reported at age 4 years: this indicates that nursery staff should look out for signs of mental health difficulties, even at this very young age. Previous research suggests that even in Norway, a country renowned for having one of the best developed health systems in the world, only 10% of 4-year olds with mental health problems receive any help, and this only increases to 25% of 10-year olds^5^: it seems likely that this figure would be higher than in other high-income countries, for example, UK and USA. On the other hand, this association between preschool and primary school mental health should also be viewed with caution: while having an abnormal score increases the odds of having later difficulties, a substantial number of children demonstrating problems at age 4 years will not be showing signs of any difficulties by age 7 years. Previous research has warned of the dangers of labelling children at the start of school, with children who were labelled to their teachers as having ADHD being *more* likely to demonstrate continuing difficulties in later primary school, in contrast to those who were displaying ADHD symptoms at the start of primary school but whose teachers were not informed that they had ADHD.^33^

Children’s backgrounds were also highlighted as important factors in the development of their mental health within the school setting. This is of key importance in areas such as Scotland, where children tend to have similar backgrounds within schools due to the catchment area system operating, in which children tend to attend the school closest to where they live.

The strongest demographic indicator of mental health difficulties was having ever had Looked After Status, similar to previous evidence which found that looked after children were more likely to have poorer mental health than children who have never had Looked After Status.^34–37^ This is likely related to the fact that the overwhelming majority of young children come into the care system because of abuse and neglect, which may well reflect an association between such adverse childhood experiences and early mental health problems.^38^ One British study found prevalence rates of any psychiatric diagnosis in looked after children in the UK as 46.4%, in contrast even to 14.6% of children in the *most disadvantaged* private households.^39^ A further study, based in Scotland, of looked after children highlighted 44% of looked after children having a psychiatric diagnosis with impaired psychosocial functioning.^35^

As this study indicated, children from the poorest backgrounds started school with higher levels of mental health problems, and the gap between the poorest and the most affluent children had increased substantially by age 7 years. It was striking that, even having controlled for the child’s initial mental health score and all other available characteristics, children living in the two poorest area deprivation quintiles had odds three times higher of having a mental health problems at age 7 years, compared with their more affluent counterparts in the top income quintile. Substantial amounts of evidence supports this association between living in poverty and poorer psychosocial outcomes during childhood.^40–42^ Multiple mechanisms are likely to be at work: children who grow up in lower income households have an increased likelihood of having parents who experience greater levels of stress, which may be transmitted to children. Furthermore, they are more likely to experience a poorer home environment, for example, having fewer resources, such as books, which may encourage optimal development, and may additionally be more likely to witness violence and other forms of domestic abuse, all of which have been evidenced to affect social, emotional and behavioural functioning.^43–45^

One hypothesis explaining this increasing disparity is that more affluent children start school ‘ahead of the game’, that is, with better language and social skills than their less affluent peers, as well as being physically taller.^46 47^ Starting school for this group may act as a stimulus, building on their prior skills and improving their social, emotional and behavioural development. Interactions between nature and nurture may also be at work; for example, children who are genetically predisposed towards hyperactivity may be more likely to exhibit symptoms when in an environment of chronic deprivation, poor peer relationships and bullying.^48^ This should be explored in future research.

## Conclusions

Children with high levels of social, emotional and behavioural problems, and those from more deprived backgrounds, need extra support in the preschool and early school years to help narrow these inequalities. Children from the most deprived backgrounds started school with higher levels of mental health difficulties, compared with the most affluent children, and this disparity widened dramatically over the first 3 years of school. Routine monitoring of the impact of primary schools on mental health is warranted.

**What is already known on this subject**

Around 1 in 10 children in the UK will experience a mental health problem. Disparities in mental health by socioeconomic group have been evidenced; however, no one knows how early they start. Schools are a prime location for intervening in mental health difficulties. At present, little is known about the impact of schools on the development of mental health in younger children and the role they play in reducing disparities between socioeconomic groups.

**What this study adds**

This study demonstrates that children from disadvantaged backgrounds start school with poorer mental health and that these disparities widen over the first 3 years of school. The majority of difference between children’s mental health development in different schools was due to differences in intake characteristics of the students.

## Footnotes

i It is important to note that the SDQ measures social, emotional and behavioural difficulties. It does not provide a mental health disorder diagnosis.

ii Cut-offs for the subscales can be found at www.sdqinfo.org

iii GIMD is produced by the Glasgow Centre for Population Health http://www.gcph.co.uk/
